# The Prognostic Value of Sarcopenia and Myosteatosis in Biliary Tract Cancer After Palliative Treatment With Radiation-Emitting Metallic Stent

**DOI:** 10.3389/fsurg.2022.852137

**Published:** 2022-04-26

**Authors:** Qi Chen, Jian Lu, Xun Lu, Xi-Juan Yao, Xuan-Pu Zhang, Shang-Yuan Wang, Jin-He Guo

**Affiliations:** ^1^Center of Interventional Radiology and Vascular Surgery, Department of Radiology, Zhongda Hospital, Medical School, Southeast University, Nanjing, China; ^2^Department of Urology, Affiliated Zhongda Hospital of Southeast University, Nanjing, China; ^3^Surgical Research Center, Institute of Urology, School of Medicine, Southeast University, Nanjing, China

**Keywords:** sarcopenia, myosteatosis, biliary tract cancer, radiation-emitting metallic stent, survival

## Abstract

**Background:**

Radiation-emitting metallic stent (REMS) placement is increasingly used for malignant biliary obstruction (MBO) caused by unresectable biliary tract carcinoma (UBTC) in clinical practice. The study is aimed to evaluate the prognostic value of sarcopenia, myosteatosis, and their combination on overall survival (OS) in patients treated with REMS for UBTC.

**Methods:**

Patients diagnosed with UBTC who underwent REMS placement between January 2013 and May 2021 were included consecutively in this retrospective study. Sarcopenia and myosteatosis were defined based on skeletal muscle index (SMI) and skeletal muscle attenuation (SMA), respectively, which were measured by computer tomography (CT) images on the level of the third lumbar vertebral body before REMS placement. Patients were categorized into two groups by sex-specific cutoff value for sarcopenia and myosteatosis, and OS rates were compared between the groups. Univariate and multivariate cox regression analyses were used to assess factors associated with OS.

**Results:**

Data of 135 patients included were retrospectively reviewed and analyzed. Median OS was 7.17 months in total cohort. Patients in the sarcopenia group had significant poorer OS than those in the non-sarcopenia group (median: 3.23 vs. 11.60 months*, p* < 0.001). OS was shorter in patients with myosteatosis than those without myosteatosis (median: 4.40 vs. 9.17 months*, p* < 0.001). Sarcopenia (odds ratio [OR] = 9.61; 95% CI = 5.41–17.09; *p* < 0.001) and myosteatosis (OR = 1.70; 95% CI = 1.13–2.57; *p* = 0.012) were significantly associated with OS. Combining sarcopenia and myosteatosis (CSM) showed a better predictive accuracy in OS than either one (area under curves: CSM vs. sarcopenia = 0.760 vs. 0.698, *p* = 0.049; CSM vs. myosteatosis = 0.760 vs. 0.671, *p* = 0.006).

**Conclusion:**

Sarcopenia and myosteatosis are negative predictors of survival in patients who underwent REMS placement for UBTC. CSM seemed to show a better prognostic value than either sarcopenia or myosteatosis alone. They can be used preoperatively for risk evaluation.

## Introduction

Biliary tract cancer (BTC) is comprised of cholangiocarcinoma (cancers located in intrahepatic, perihepatic, and distal bile duct), gallbladder carcinoma, and ampulla of Vater carcinoma. BTC accounts for <1% of all tumors, with a deplorable 5-year survival rate of <10% (1). Surgical resection remains the cornerstone of management in early-stage BTC (2). For metastatic or unresectable BTC (UBTC), jaundice caused by malignant biliary obstruction (MBO) frequently occurs (3), which is commonly resolved with palliative treatment with percutaneous transhepatic biliary stent (PTBS) placement. Previous studies have demonstrated that radiation-emitting metallic stent (REMS) in patients with MBO can improve the patency of stent and provide a better survival than the insertion of a conventional self-expand metallic stent (SEMS) (4–9). Many studies have explored factors relevant to overall survival (OS) in patients with BTC after certain management modalities, such as surgery, systemic therapy, or conventional biliary stenting (10–12). Moreover, with regard to prognostic factors, several studies have mainly focused on tumor-related parameters, for instance, tumor node metastasis (TNM) stage, tumor number, and tumor diameter (4, 13).

In recent years, a disease named sarcopenia has aroused attention, which was first proposed by Rosenberg to describe skeletal muscle mass depletion in 1989 (14). At first, sarcopenia is only considered an age-related disease, since skeletal muscle mass diminishes at a rate of 8–15% every 10 years after the age of 40 years (15). However, now it is generally considered to be an active and systemic reduction of skeletal muscle mass and strength (16), which can be caused by malnutrition, endocrine disorders, chronic inflammatory diseases (such as tumors), etc., and can be met at any age (17). Myosteatosis is defined as the form of low skeletal muscle attenuation (SMA) measured by computer tomography (CT) in a cross-section slice on the level of the third lumbar vertebral body (L3), which can represent reduced muscle function and strength (12). A number of factors were elucidated to impact attenuated muscle density, which included obesity, male gender, increasing age, type 2 diabetes mellitus, inactivity, malignancies, and host systemic inflammatory response (18). Many studies have confirmed that sarcopenia has negative effects on treatment efficacy and OS in many malignancies, such as cholangiocarcinoma (10), gallbladder cancer (15), renal cancer (19), bladder cancer (20), and hepatic malignancies (21). Likewise, myosteatosis is significantly associated with postoperative mortality in various kinds of malignancies (12, 17). In BTC setting, there are many studies exploring the relationship between body compositions (such as sarcopenia and/or myosteatosis) and prognosis in patients limited to a specific cancer location (i.e., intrahepatic cholangiocarcinoma, perihilar cholangiocarcinoma, distal bile duct cancer, gallbladder cancer, or a combination of facultative BTC) or those who underwent particular treatment strategies (i.e., surgery, chemotherapy, and palliative methods such as PTBS) (10–12, 15, 17, 18). To the best of our knowledge, there are few studies that analyze the effect of sarcopenia combined with myosteatosis on survival in patients following PTBS placement by integrating all BTCs, especially following REMS placement.

This retrospective study is aimed to investigate the impact of body compositions (sarcopenia and myosteatosis) on survival in patients with UBTC who underwent REMS placement and attempt to establish a new combination score to predict mortality in those patients above mentioned.

## Patients and Methods

### Patients and Data Collection

In this study, patients with UBTC who underwent REMS placement for palliative treatment between January 2013 and May 2021 at the authors' center were retrospectively included. The inclusion criteria were as follows: 1) age older than 18; 2) diagnosed with BTC, such as cholangiocarcinoma, gallbladder carcinoma, and ampulla of Vater carcinoma; 3) patients with unresectable lesions or refused surgical treatment; and 4) with symptoms of jaundice due to MBO. The exclusion criteria were as follows: 1) with the previous history of stenting or surgery; 2) with additional concurrent malignant tumor; and 3) without preoperative CT images containing L3 level or absence of complete data.

Patients were regularly followed up at 1 week, every month at the first 6 months, and then every 3 months after REMS placement. All data included were reviewed and collected from electronic medical records. These data included demographics, history of diabetes mellitus, history of hepatitis B virus (HBV), preoperative body mass index (BMI), carbohydrate antigen 19-9 (CA 19-9), total bilirubin (TB), albumin, hemoglobin, SMA, skeletal muscle index (SMI), Eastern Cooperative Oncology Group (ECOG) performance-status score, Child-Pugh score, tumor etiology, the extent of disease, and adjuvant therapy.

### Treatment Protocol

The REMS is composed of two parts, i.e., an inner uncovered SEMS and an outer stent containing ^125^I seed (CIAE-6711; Chinese Atomic Energy Science Institution, Beijing, China). Numbers, prescription dose, and distribution of ^125^I seed were planned and calculated according to the Treatment Planning System (TPS, FTT Technology, Beijing, China), as described in our previous study (4). Prior to REMS operation, ^125^I seeds were preloaded into the surface of the outer stent. The dilated hepatic bile duct was punctured percutaneously under ultrasound or fluoroscopy, and the location of stricture was confirmed *via* cholangiogram. After that, physicians dilated the passage with a balloon dilator catheter, exchanged a stiff guidewire, and deployed the outer ^125^I seed-loaded stent into the targeted location through a 10 French sheath. In addition, then immediately an inner uncovered SEMS (Nanjing Micro-Tech Co. Ltd., Nanjing, China) was implanted to cover the outer stent. External drainage catheter (Cook Medical, Bloomington, Indiana, USA) was routinely placed above the level of the diseased bile duct to prevent dysfunction of stent caused by possible bleeding or sludging and to provide convenience for the secondary operation just in case. When the operation was evaluated clinically successful 1 week later, the catheter would be pulled out. Subsequent adjuvant local treatment and systemic therapy were permitted.

### Outcomes and Definitions

**Figure 1:**
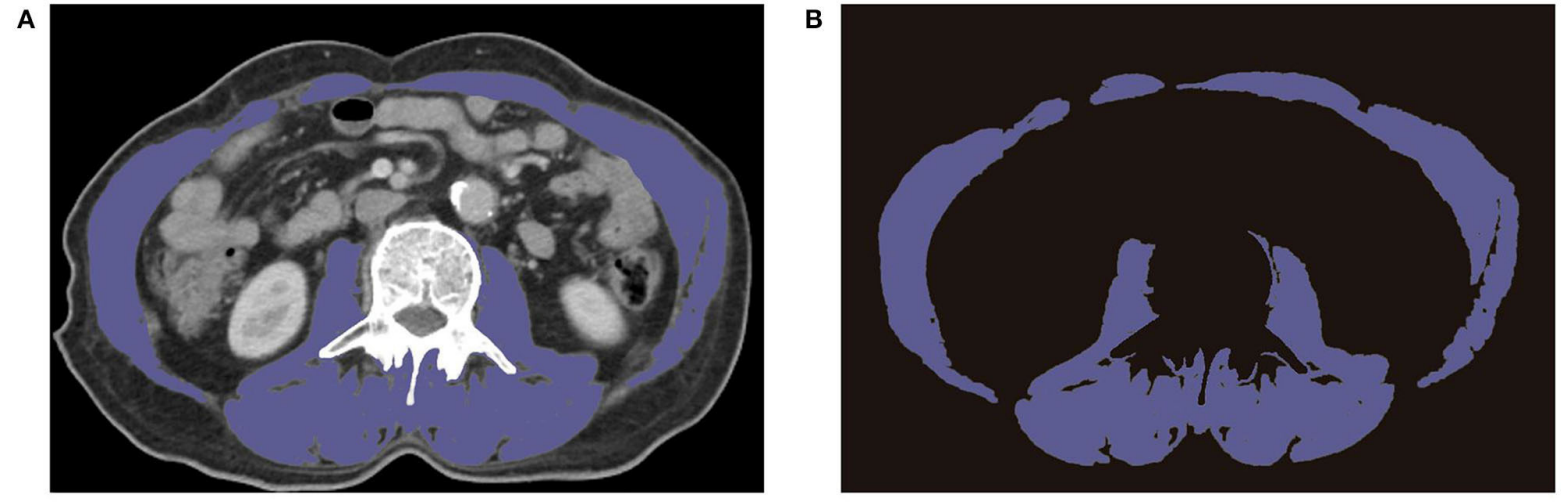
Preoperative CT images of L3 region showing the border of skeletal muscle area. **(A)** Representative CT images on the level of L3. **(B)** Extracted images of skeletal muscle on the level of L3.

CT images in the venous phase on the level of the mid-L3 vertebral body were captured to delineate skeletal muscle area within 1 month before REMS placement. The skeletal muscle areas included the rectus abdominis, transverse abdominal, internal oblique, external oblique, paraspinal muscle, and psoas. The skeletal muscle area was chosen in a semi-automated method, with a manual outlining the skeletal muscle border (Figure 1). SMIs were obtained by normalization following this formula: SMI = skeletal muscle area (cm^2^)/the square of the height (m^2^). SMAs were determined by calculating the average Hounsfield unit (HU) of skeletal muscle area, except for intermuscular adipose tissue. Considering the different races and diseases in diverse studies, the threshold of SMI and SMA was determined according to this study population. SMI lower than the cutoff was defined as sarcopenia, likewise, SMA lower than cutoff was labeled as myosteatosis. The end-point OS was defined from the date of REMS placement to death or the last follow-up.

### Statistical Analysis

Continuous variables were presented as mean ± standard deviation (SD) or medians with interquartile range (IQR), as appropriate. Categorical variables were expressed as frequency and percentages. Characteristics and variables between the groups were compared using a two-sample independent *t*-test or Mann-Whitney test for numerical data, and Pearson's chi-square or Fisher's exact tests for categorical data. OS was calculated using the Kaplan-Meier (K-M) method, and differences between the curves were compared by log-rank test. Receiver operating characteristic (ROC) curve analysis was used to identify the cutoff value of sarcopenia and myosteatosis. Univariate and multivariable analyses were used to evaluate the significant predictors of OS. Spearman's correlation analysis was used for correlation analysis. The area under the receiver operating characteristic curve (AUROC) was calculated to access and compare the prognostic value of sarcopenia and myosteatosis. Statistical analysis was performed using the software Stata 15.1 (StataCorp, College Station, TX, USA). *p* < 0.05 was considered statistically significant.

## Results

### Baseline Characteristics

**Figure 2:**
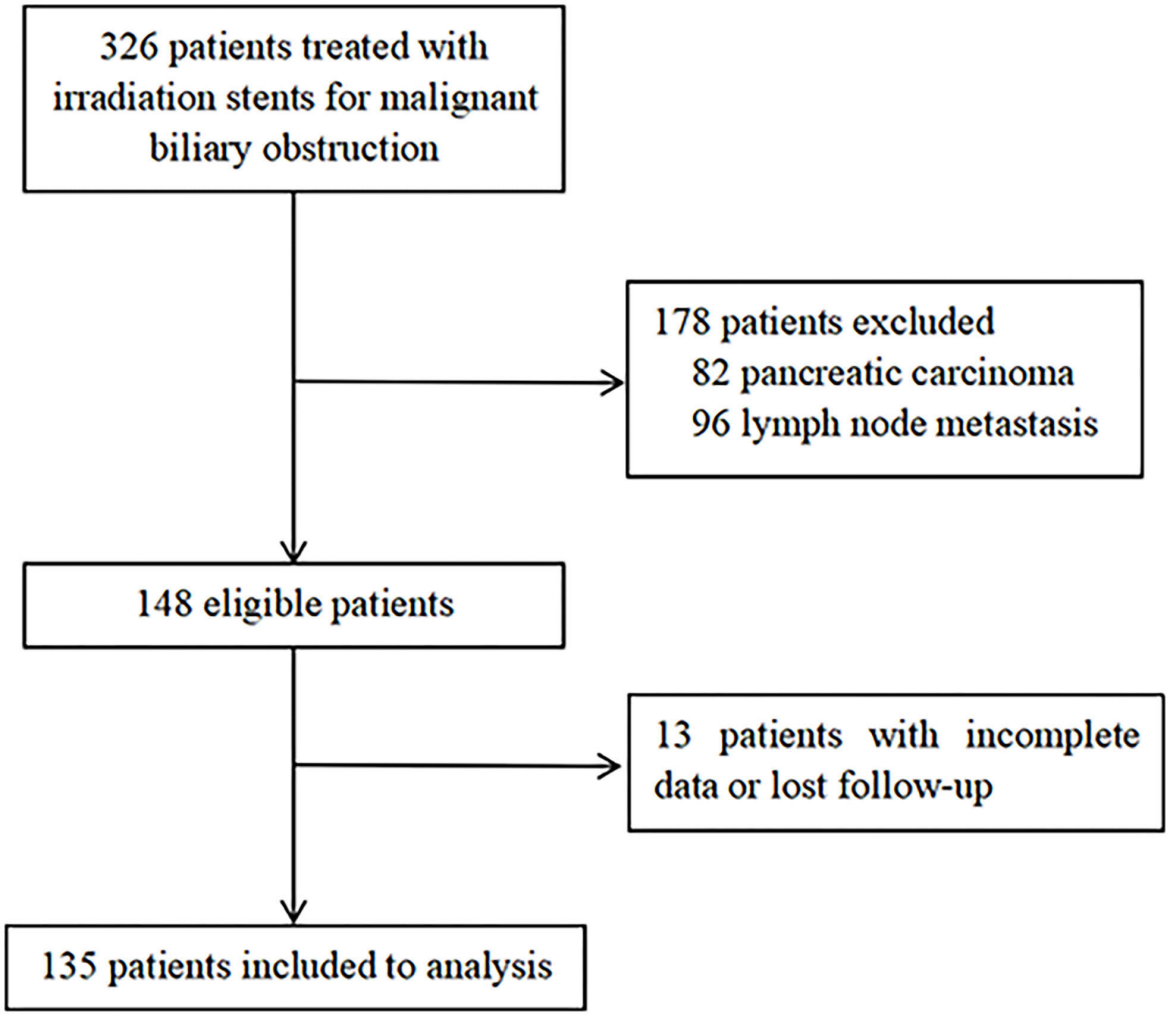
The flow diagram of patient selection.

A total of 326 patients in the authors' center treated with REMS for MBO in UBTC were screened (Figure 2), of which 135 patients were included in this study. There were 69 (51.1%) men and 66 (48.9%) women, and the median (IQR) age was 71 (61–79) years. The tumor etiologies were cholangiocarcinoma in 102 (75.6%) patients, gallbladder carcinoma in 28 (20.7%) patients, and ampulla of Vater carcinoma in 5 (3.7%) patients. The distribution of ECOG performance-status score of 0, 1, 2, 3 was in 12 (8.9%) patients, 71 (52.6%) patients, 29 (21.5%) patients, and 23 (17.0%) patients, respectively. The extent of disease was locally advanced and metastatic in 120 (88.9%) patients and 15 (11.1%) patients. Forty-six (34.1%) patients experienced adjuvant therapy. Baseline characteristics of the patients included are shown in Table 1.

**Table 1:** Baseline characteristics of included patients.

**Variable**	**Result**
Total, *n*	135
Age, years, median (IQR)	71 (61–79)
Gender, *n* (%)	
Male	69 (51.1%)
Female	66 (48.9%)
BMI, Kg/m^2^, median (IQR)	20.8 (19.0–23.3)
SMA, HU, median (IQR)	38.1 (30.1–46.0)
SMI, cm^2^/m^2^, median (IQR)	41.6 (35.4–48.7)
CA19-9 level, U/mL, median (IQR)	315.8 (108.5–1000.0)
Total bilirubin level, μmol/L, median (IQR)	97.4 (53.0–185.2)
Albumin, g/L, median (IQR)	34.2 (31.9–37.7)
Hemoglobin, g/L, mean ± SD	110.9 ± 17.1
Diabetes mellitus, *n* (%)	
No	110 (81.5%)
Yes	25 (18.5%)
History of HBV, *n* (%)	
No	125 (92.6%)
Yes	10 (7.4%)
ECOG performance-status score, *n* (%)	
0	12 (8.9%)
1	71 (52.6%)
2	29 (21.5%)
3	23 (17.0%)
Child-Pugh score, *n* (%)	
A	55 (40.7%)
B	80 (59.3%)
Tumor etiology, *n* (%)	
Cholangiocarcinoma	102 (75.6%)
Gallbladder carcinoma	28 (20.7%)
AOV carcinoma	5 (3.7%)
Extent of disease, *n* (%)	
Locally advanced	120 (88.9%)
Metastatic	15 (11.1%)
Adjuvant therapy, *n* (%)	
No	89 (65.9%)
Yes	46 (34.1%)

*BMI, body mass index; SMA, skeletal muscle attenuation; SMI, skeletal muscle index; CA, carbohydrate antigen; HBV, hepatitis B virus; ECOG, Eastern Cooperative Oncology Group; AOV, ampulla of Vater*.

### Cutoff of Sarcopenia and Myosteatosis Measured by SMI and SMA

**Figure 3:**
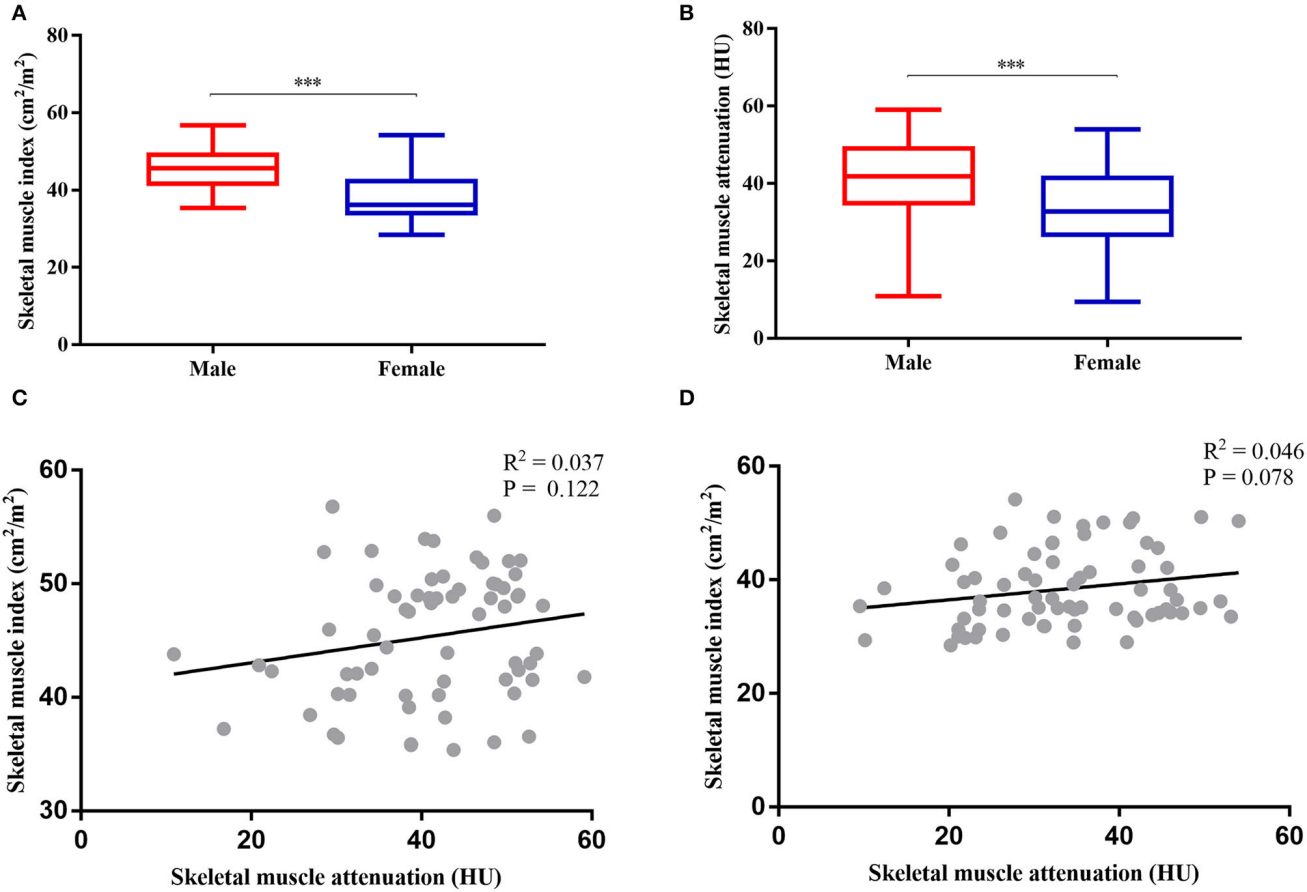
Gender-specific body compositions and their correlation. **(A)** Comparison between skeletal muscle index (SMI) and sex. **(B)** Comparison between skeletal muscle attenuation (SMA) and sex. **(C)** Correlation between SMI and SMA in women. **(D)** Correlation between SMI and SMA in men. ***P < 0.001.

**Figure 4:**
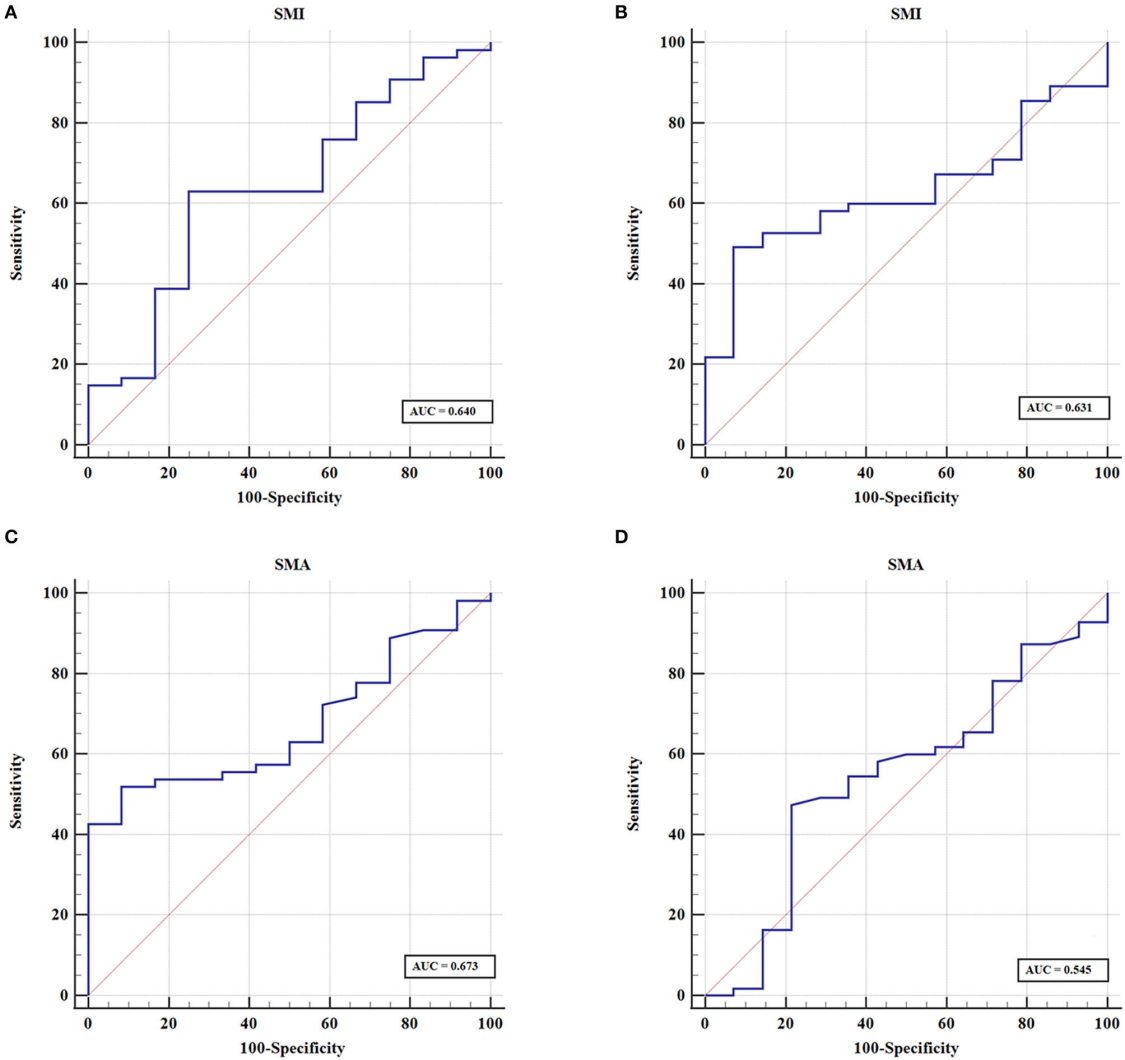
Sex-specific cutoff values established for skeletal muscle index (SMI) and skeletal muscle attenuation (SMA) using ROC curves. **(A)** The optimal cutoff of SMI value in men. **(B)** The optimal cutoff of SMI value in women. **(C)** The cutoff value for SMA in men. **(D)** The cutoff value for SMA in women.

Sarcopenia and myosteatosis were defined on the basis of the values of SMI and SMA. SMI and SMA were both significantly higher in men than in women (*p* < 0.001; Figures 3A,B). Moreover, we found no significant correlation between SMI and SMA (Figures 3C,D). Therefore, the sex-specific threshold for SMI and SMA was established. The optimal cutoff values of SMI in men and women were 47.72 and 35.00 cm^2^/m^2^, and the cutoff values for SMA in men and women were 40.88 and 31.23 HU (Figures 4A–D). Patients with SMI and SMA lower than the cutoff values were labeled as sarcopenia and myosteatosis.

### Comparison of Characteristics Between Patients With and Without Sarcopenia

Patients in this cohort were divided into two groups based on the existence of sarcopenia. There were 64 (47.4%) patients in the sarcopenia group and 71 (52.6%) patients in the non-sarcopenia group. The comparison of baseline characteristics between the two groups is demonstrated in Table 2. There were more men in the sarcopenia group than in the non-sarcopenia group (57.8 vs. 40.8%, *p* = 0.049). Individuals with sarcopenia were on average with poorer ECOG performance-status scores than those without sarcopenia (ECOG 2: 28.1 vs. 7.0%; ECOG 3: 32.9 vs. 11.3%, *p* < 0.001). Child-Pugh B scores were more common in individuals with sarcopenia when compared with those without (68.7 vs. 50.7%, *p* = 0.033). More patients without sarcopenia received adjuvant therapy than those with sarcopenia (50.7 vs. 15.6%, *p* < 0.001).

**Table 2:** Comparison between sarcopenia and non-sarcopenia group when using SMI as an assessment tool.

	**SMI**		
**Variable**	**Sarcopenia**	**Non-sarcopenia**	***P*-value**
Total, *n* (%)	64 (47.4%)	71 (52.6%)	
Age, years, median (IQR)	70 (60.8–78.3)	71 (61.5–79.0)	0.700
Gender, *n* (%)			**0.049**
Female	27 (42.2%)	42 (59.2%)	
Male	37 (57.8%)	29 (40.8%)	
BMI, Kg/m^2^, median (IQR)	20.5 (18.7–23.0)	21.1 (19.1–23.4)	0.377
CA19-9, U/mL, median (IQR)	466.3 (115.0–1000.0)	225.0 (97.4–1000.0)	0.359
TB, μmol/L, median (IQR)	123.7 (64.2–186.5)	73.4 (42.6–172.5)	0.056
ALB, g/L, median (IQR)	33.6 (31.9–36.8)	35.0 (32.0–38.4)	0.087
Hemoglobin, g/L, mean ± SD	109.3 ± 17.1	112.3 ± 17.2	0.308
Diabetes mellitus, *n* (%)			0.948
No	52 (81.2%)	58 (81.7%)	
Yes	12 (18.8%)	13 (18.3%)	
History of HBV, *n* (%)			0.865
No	59 (92.2%)	66 (93.0%)	
Yes	5 (7.8%)	5 (7.0%)	
ECOG, *n* (%)			**<0.001**
0	2 (3.1%)	10 (14.1%)	
1	23 (35.9%)	48 (67.6%)	
2	21 (32.9%)	8 (11.3%)	
3	18 (28.1%)	5 (7.0%)	
Child-Pugh score, *n* (%)			**0.033**
A	20 (31.3%)	35 (49.3%)	
B	44 (68.7%)	36 (50.7%)	
Tumor etiology, *n* (%)			0.462
Cholangiocarcinoma	48 (75.0%)	54 (76.1%)	
Gallbladder carcinoma	15 (23.4%)	13 (18.3%)	
AOV carcinoma	1 (1.6%)	4 (5.6%)	
Extent of disease, *n* (%)			0.300
Locally advanced	55 (85.9%)	65 (91.5%)	
Metastatic	9 (14.1%)	6 (8.5%)	
Adjuvant therapy, *n* (%)			**<0.001**
No	54 (84.4%)	35 (49.3%)	
Yes	10 (15.6%)	36 (50.7%)	

*SMI, skeletal muscle index; BMI, body mass index; CA, carbohydrate antigen; TB, total bilirubin; ALB, albumin; HBV, hepatitis B virus; ECOG, Eastern Cooperative Oncology Group; AOV, ampulla of Vater. All bold values are of significant difference*.

### Comparison of Characteristics Between Patients With and Without Myosteatosis

All the patients were classified into the myosteatosis group and the non-myosteatosis group, there were 58 (43.0%) patients in the myosteatosis group and 77 (57.0%) patients in the non-myosteatosis group. Comparison of baseline characteristics between these two groups is summarized in Table 3. Patients in the myosteatosis group were older than those in the non-myosteatosis group [75 (66–81) vs. 68 (57–76), *p* = 0.005]. The proportion of patients with Child-Pugh B score and receiving non-adjuvant therapy was higher in the myosteatosis group than the non-myosteatosis group (Child-Pugh B score: 69.0 vs. 51.9%, *p* = 0.046; adjuvant therapy: 84.5 vs. 51.9%, *p* < 0.001). However, no statistically significant difference was met in gender and ECOG (gender, *p* = 0.823; ECOG, *p* = 0.417) when myosteatosis was used as an assessment tool.

**Table 3:** Comparison between myosteatosis and non-myosteatosis group when using SMA as an assessment tool.

	**SMA**		
**Variable**	**Myosteatosis**	**Non-myosteatosis**	***P*-value**
Total, *n* (%)	58 (43.0%)	77 (57.0%)	
Age, years, median (IQR)	75 (66–81)	68 (57–76)	**0.005**
Gender, *n* (%)			0.823
Female	29 (50.0%)	40 (51.9%)	
Male	29 (50.0%)	37 (48.1%)	
BMI, Kg/m^2^, median (IQR)	21.4 (19.1–23.8)	20.8 (16.7–22.4)	0.111
CA19-9, U/mL, median (IQR)	359.0 (117.8–1000.0)	310.0 (88.4–1000.0)	0.530
TB, μmol/L, median (IQR)	125.9 (63.8–193.0)	85.8 (40.0–176.2)	0.053
ALB, g/L, median (IQR)	33.9 (32.2–36.6)	34.5 (31.8–38.2)	0.241
Hemoglobin, g/L, mean ± SD	109.3 ± 17.1	112.1 ± 17.2	0.366
Diabetes mellitus, *n* (%)			0.312
No	45 (77.6%)	65 (84.4%)	
Yes	13 (22.4%)	12 (15.6%)	
History of HBV, *n* (%)			0.389
No	55 (94.8%)	70 (90.9%)	
Yes	3 (5.2%)	7 (9.1%)	
ECOG, *n* (%)			0.417
0	3 (5.2%)	9 (11.7%)	
1	29 (50.0%)	42 (54.5%)	
2	14 (24.1%)	15 (19.5%)	
3	12 (20.7%)	11 (14.3%)	
Child-Pugh score, *n* (%)			**0.046**
A	18 (31.0%)	37 (48.1%)	
B	40 (69.0%)	40 (51.9%)	
Tumor etiology, *n* (%)			0.663
Cholangiocarcinoma	42 (72.4%)	60 (77.9%)	
Gallbladder carcinoma	13 (22.4%)	15 (19.5%)	
AOV carcinoma	3 (5.2%)	2 (2.6%)	
Extent of disease, *n* (%)			0.759
Locally advanced	51 (87.9%)	69 (89.6%)	
Metastatic	7 (12.1%)	8 (10.4%)	
Adjuvant therapy, *n* (%)			**<0.001**
No	49 (84.5%)	40 (51.9%)	
Yes	9 (15.5%)	37 (48.1%)	

*SMA, skeletal muscle attenuation; BMI, body mass index; CA, carbohydrate antigen; TB, total bilirubin; ALB, albumin; HBV, hepatitis B virus; ECOG, Eastern Cooperative Oncology Group; AOV, ampulla of Vater. All bold values are of significant difference*.

### OS Analysis

**Figure 5:**
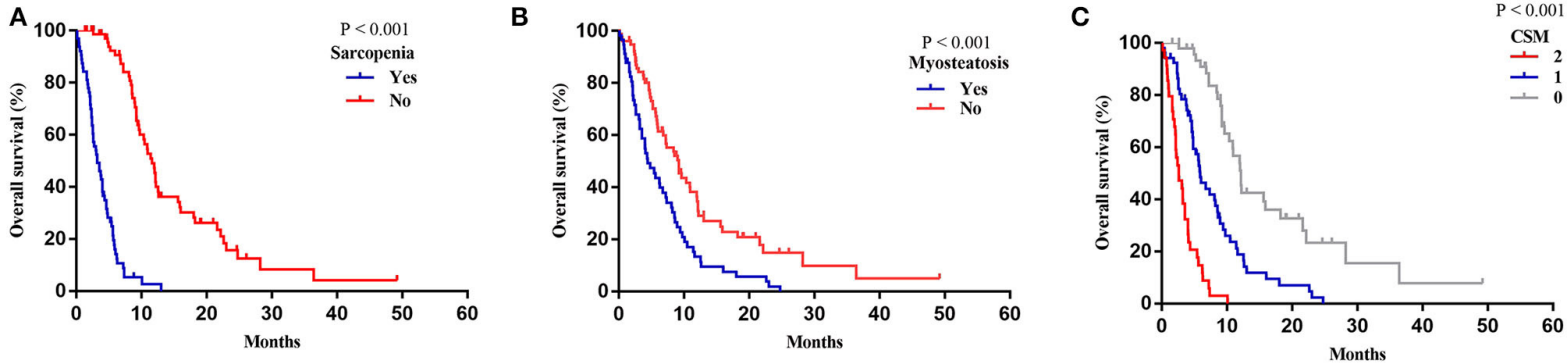
Overall survival based on sarcopenia, myosteatosis, and CSM. **(A)** Sarcopenia. **(B)** Myosteatosis. **(C)** CSM.

The median follow-up time was 24.6 (IQR, 12.27–49.17) months, and the median OS of the patients following REMS placement was 7.17 months. The OS curves for patients with sarcopenia and myosteatosis are illustrated in Figures 5A,B. Patients with sarcopenia showed a significantly shorter OS than those without sarcopenia (median: 3.23 vs. 11.60 months, *p* < 0.001; Figure 5A). Likewise, patients with myosteatosis showed a significantly shorter OS than those without myosteatosis (median: 4.40 vs. 9.17 months, *p* < 0.001; Figure 5B).

### Univariate and Multivariate Analyses for OS

Univariate analysis showed that myosteatosis [odds ratio (OR), 2.02; 95% confidence interval (CI), 1.38–2.95, *p* < 0.001], ECOG 1 (OR, 2.07; 95% CI, 0.94–4.57, *p* = 0.071), ECOG 2 (OR, 4.41; 95% CI, 1.88–10.37, *p* = 0.001), ECOG 3 (OR, 5.11; 95% CI, 2.13–12.26, *p* < 0.001), sarcopenia (OR, 8.87; 95% CI, 5.54–14.19, *p* < 0.001), gallbladder carcinoma (OR, 1.61; 95% CI, 1.02–2.57, *p* = 0.043), Child-Pugh B score (OR, 1.61; 95% CI, 1.09–2.38, *p* = 0.016), presence of adjuvant therapy (OR, 0.52; 95% CI, 0.35–0.79, *p* = 0.002), and presence of metastases (OR, 2.29; 95% CI, 1.27–4.13, *p* = 0.006) were significantly related to OS (Table 4). After incorporating all the above variables into multivariate analysis, myosteatosis (OR, 1.70; 95% CI, 1.13–2.57, *p* = 0.012), ECOG 1 (OR, 2.31; 95% CI, 1.01–5.30, *p* = 0.049), ECOG 2 (OR, 2.52; 95% CI, 1.00–6.34, *p* = 0.049), ECOG 3 (OR, 2.90; 95% CI, 1.10–7.62, *p* = 0.031), sarcopenia (OR, 9.61; 95% CI, 5.41–17.09, *p* < 0.001), gallbladder carcinoma (OR, 1.81; 95% CI, 1.10–2.97, *p* = 0.020), Child-Pugh B score (OR, 2.03; 95% CI, 1.33–3.08, *p* = 0.001), and presence of metastases (OR, 2.46; 95% CI, 1.30–4.64, *p* = 0.006) were demonstrated to be independent predictors of OS (Table 4).

**Table 4:** Univariate and multivariate cox regression analyses for overall survival.

	**Univariate analysis**			**Multivariate analysis**		
**Variable**	**OR**	**95%CI**	***P***	**OR**	**95%CI**	***P***
Age	0.99	0.98–1.01	0.374			
Gender	1.08	0.74–1.57	0.697			
Myosteatosis	2.02	1.38–2.95	<0.001	1.70	1.13–2.57	0.012
BMI	1.01	0.95–1.07	0.782			
ECOG						
1	2.07	0.94–4.57	0.071	2.31	1.01–5.30	0.049
2	4.41	1.88–10.37	0.001	2.52	1.00–6.34	0.049
3	5.11	2.13–12.26	<0.001	2.90	1.10–7.62	0.031
Sarcopenia	8.87	5.54–14.19	<0.001	9.61	5.41–17.09	<0.001
Etiology						
Gallbladder	1.61	1.02–2.57	0.043	1.81	1.10–2.97	0.020
AOV	0.54	0.17–1.77	0.309	0.51	0.15–1.70	0.271
CA19-9 level	1.00	0.99–1.00	0.321			
Child-Pugh score	1.61	1.09–2.38	0.016	2.03	1.33–3.08	0.001
Adjuvant therapy	0.52	0.35–0.79	0.002	1.11	0.68–1.79	0.679
History of HBV	0.93	0.41–2.13	0.863			
Tumor extent	2.29	1.27–4.13	0.006	2.46	1.30–4.64	0.006
ALB level	0.98	0.94–1.02	0.251			
Hemoglobin	0.99	0.99–1.01	0.782			
Diabetes	1.03	0.64–1.66	0.915			

*BMI, body mass index; ECOG, Eastern Cooperative Oncology Group; AOV, ampulla of Vater; CA, carbohydrate antigen; HBV, hepatitis B virus; ALB, albumin*.

**Figure 6:**
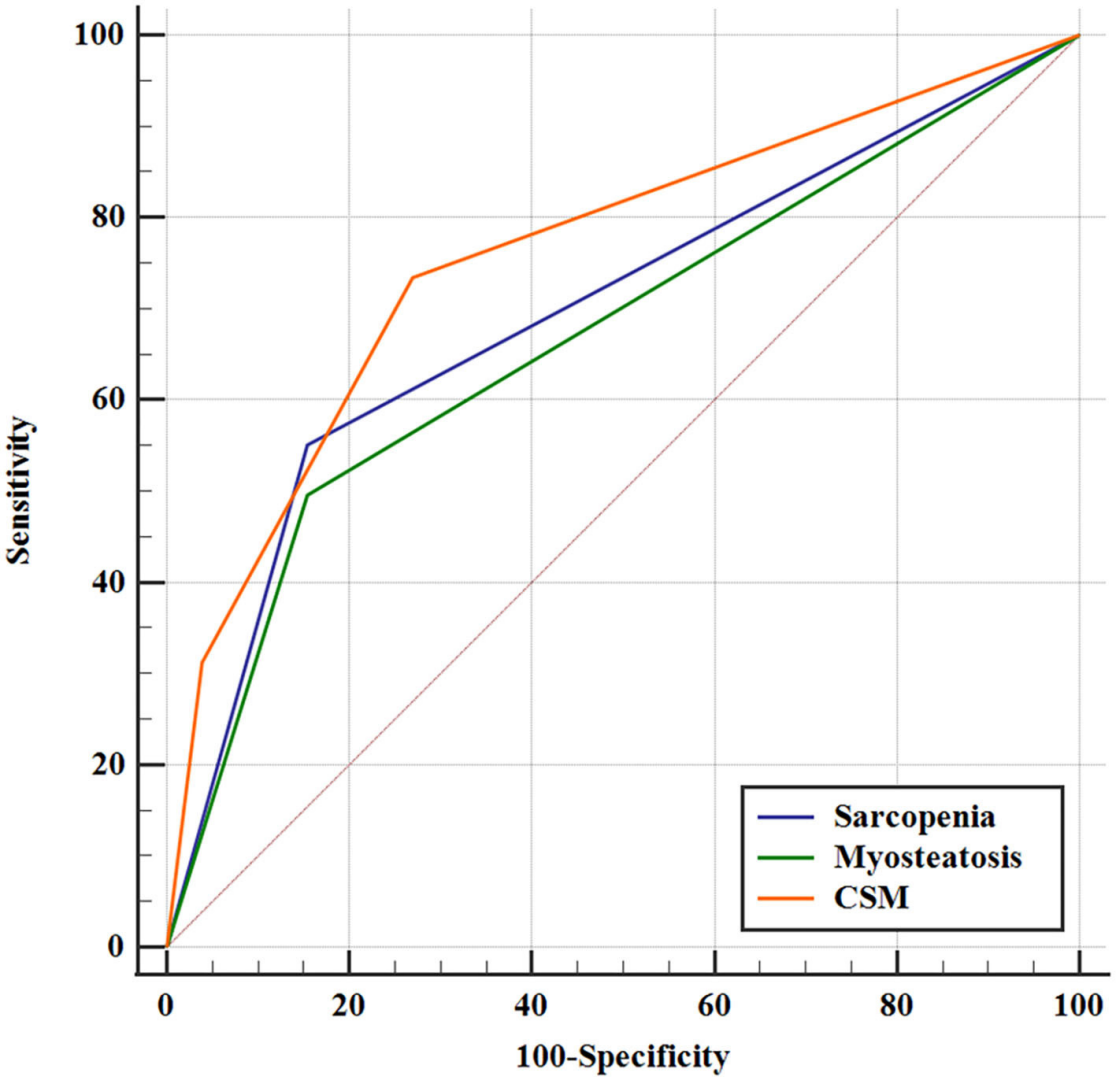
The predictive value of sarcopenia, myosteatosis, and CSM by ROC curves.

A more precise model combining sarcopenia and myosteatosis (CSM) was created to explore the effect of sarcopenia and myosteatosis. Each variable was endowed with one score. The area under curve (AUC) values of models based on sarcopenia, myosteatosis, and CSM were 0.698, 0.671, and 0.760, respectively (Table 5). Moreover, the comparison of ROC curves showed that CSM model was better than sarcopenia (*p* = 0.049) and myosteatosis (*p* = 0.006) model, and there was no significant difference between the sarcopenia and myosteatosis models (*p* = 0.631; Figure 6). Stratified by the CSM model, patients with sarcopenia and myosteatosis were classified into CSM 2 group. Patients with non-sarcopenia and non-myosteatosis were in CSM 0 group. The remaining patients with either one were classified into CSM 1 group. We compared the differences in OS between distinct CSM groups. The K-M curves demonstrated that the median OS in CSM 0 group was 12.10 months, 5.87 months in CSM 1 group, and 2.63 months in CSM 2 group. OS was the highest in CSM 0 group and the lowest in the CSM 2 group (*p* < 0.001; Figure 5C).

**Table 5:** The AUCs of sarcopenia, myosteatosis, and CSM for OS and prediction based on the ROC results.

	**OS**		
**Variable**	**AUC**	**95% CI**	***P*-value**
Sarcopenia	0.698	0.613–0.774	<0.001
Myosteatosis	0.671	0.585–0.749	<0.001
CSM	0.760	0.679–0.829	<0.001

*AUCs, area under curves; CSM, combining sarcopenia and myosteatosis; OS, overall survival; ROC, Receiver operating characteristic*.

## Discussion

The present study elucidated that body compositions (sarcopenia and myosteatosis based on SMI and SMA, respectively) measured by axial CT scan images at the L3 level were significant predictive factors on mortality in patients with BTC that underwent REMS placement. It was further confirmed that CSM score combining sarcopenia and myosteatosis was a new host-related biomarker to predict survival in patients with BTC following REMS placement. Based on our best knowledge, this is the first study to focus on patient-specific parameters and investigate the impact of these parameters on survival in patients with BTC after palliative treatment with REMS.

Since it was proposed in 1989, sarcopenia has been used not only as age-related variable but also as a variable caused by different diseases, such as malignant tumors (17). European Working Group on sarcopenia in the elderly (EWGSOP) proposes the definition of sarcopenia as the progressive and widespread loss of skeletal muscle mass and strength, which has been widely used all over the world (22). Skeletal muscle mass on the level of L3 vertebra strongly correlates to total skeletal tissue mass (23), which needs to index with the stature to generate SMI, distinguishing sarcopenia and non-sarcopenia. In recent years, sarcopenia is recognized as a disease entity with its own the International Classification of Diseases (ICD)-10 code (M62.84) (24). Many studies demonstrated that sarcopenia was related to an increased risk of postoperative morbidity and mortality (12). Myosteatosis is a reflection of muscle quality, portending altered intramyocellular triglycerides, muscle edema, conversion in muscle structure, and disorders of regulation of the host systemic inflammatory response (25), which is related to a dismal prognosis in cancer patients (17). Sarcopenia and myosteatosis are assessed at the level of L3 on a basis of CT scan, which is routinely examined in cancerous patients to evaluate location and stage of malignancy, thus, there is no financial burden to these patients.

In the present study, the sex-specific threshold of SMI and SMA was utilized to define sarcopenia and myosteatosis in this study population. Although some cutoff values for distinguishing sarcopenia and myosteatosis have been reported in cancer settings (26–28), there is still no definite criterion for the differentiation of sarcopenia and myosteatosis. In Asian countries, the Japan Society of Hepatology (JSH) guidelines of sarcopenia were defined as L3 muscle index <42 cm^2^/m^2^ for male and 38 cm^2^/m^2^ for female patients (29). Due to different sex, ethnicity, and baseline characteristics in diverse cancers, we set up our sex-specific cutoffs, where sarcopenia was restricted to L3 SMI <47.72 for male patients and 35.00 cm^2^/m^2^ for female patients. The optimal cutoff values for SMA in men and women were 40.88 and 31.23 HU, respectively, in our research population. The optimal threshold of certain cancers used worldwide is yet to be established.

In the present study, there are more men, poor ECOG performance-status score patients, Child-Pugh B score patients, and receiving supportive treatment patients in the sarcopenia group than non-sarcopenia one. Moreover, patients with myosteatosis are much older, more in Child-Pugh B score, and tend to receive no adjuvant therapy, when compared to those in non-myosteatosis group. Previous study has reported that inter- and intramuscular lipid deposition were increased with increasing age (30), moreover, there is evident relationship between sex and skeletal muscle mass (31), which is consistent with our results. We postulate that sarcopenia and myosteatosis mean poor physiological reserve and resilience in patients. When patients are diagnosed with both two or either one, they are vulnerable to have worse ECOG performance status, worse Child-Pugh score, and tend to not tolerate or refuse further adjuvant treatment due to personal physical reasons. The performances status, such as ECOG performance-status, is host related to assess physiologic reserves, however, does not present a quantitative condition of patients' preoperative general condition because of subjectivity and imprecision (15). Sarcopenia and myosteatosis offset the limitation and can be used to reflect the physiological reserve objectively and quantitatively (32).

Sarcopenia and myosteatosis are demonstrated to be independently significant predictors of mortality in patients with BTC following REMS placement in our study. Yoon et al. investigated body compositions in patients with BTC who underwent curative surgery, and a total of 371 patients were incorporated into analysis, which clarified that Low SMI and low SMA were independent prognostic factors of survival, and the combination of high SMI and high SMA had the best prognosis (12). Our results showed that sarcopenia and myosteatosis based on SMI and SMA were independent risk factors of patients with BTC following REMS placement, and combining these two parameters exhibited a better prognostic value than either one, which is similar to Yoon's research. A systematic review confirmed that sarcopenia identified before surgery with the aid of CT was associated with dismal OS in gastrointestinal and hepato-pancreato-biliary malignancies (33). With regard to biliary stenting, Zhang et al. (11) reported that low SMI was a negative predictor of OS after PTBD for patients with perihilar cholangiocarcinoma, which was in accord with our study. Our study incorporated all kinds of BTC beyond only perihilar cholangiocarcinoma, which further confirmed the predictive value of sarcopenia in BTC. We believe that future prospective analysis of body compositions in certain cancer and definite treatment is needed.

There are many elements influencing skeletal muscle mass. Clinically, anorexia, malabsorption, indigestion, and dysphagia related to malignant disease, especially advanced stage, result in decreasing nutrients and energy, causing depletion of muscle mass (34). Some studies have shown that skeletal muscle can produce hormones that are labeled as myokines, which are inclined to downgrade the levels of pro-inflammatory cytokines and growth factors, which have been demonstrated to work crucially in the process of tumor progression, such as tumor necrosis factor-a, insulin-like growth factor-I, and leptin (35–37). We can speculate that sarcopenia caused by muscle loss in cancer patients can bring on the decreased secretion of myokines, ultimately leading to cancer progression. Myosteatosis caused by infiltration of lipids into both the inter-and intramyocellular compartments has been demonstrated to be relevant to the presence of host systemic inflammation (31). Some studies have assumed that penetration of lipids into myocellular compartments has overall toxic effects and leads to insulin resistance, potentially aggravating systemic inflammation in reverse (38). However, the pathophysiological mechanism is very complicated and cannot be fully stated, which needs to be investigated in future days.

Interestingly, this study reveals that patients with CSM 2 score based on sarcopenia combined with myosteatosis have poorest survival, with median survival of 2.63 months, which suggests that excessive operation should be averted when patients are diagnosed with sarcopenia accompanied by myosteatosis. Many studies suggested that interventional rehabilitation before or after surgery that includes nutrition support and physical exercise should be put in practice to improve clinical outcome (39). Some studies explored that non-steroidal anti-inflammatory medication should be used to regulate host-related systemic inflammation (40, 41). A prospective study should be conducted to prove the effect of rehabilitation management in certain patients.

There are some limitations to this study. Firstly, because of its single-experience, retrospective nature, and exclusion of patients due to incomplete clinical data, selection bias cannot be avoided. Thus, further prospective clinical studies are required to be conducted to strengthen the level of evidence. Secondly, skeletal muscle area was measured in a semi-automated method, with a manual outlining skeletal muscle border, resulting in ineluctable measurement deviation. Thirdly, this study lacks the analysis of other body compositions (such as visceral and subcutaneous adipose tissue areas), the fluctuation of body compositions, and other end-point (such as patency of stent and jaundice remission rate), which can be evaluated in the future studies.

## Conclusion

This retrospective study demonstrated that sarcopenia and myosteatosis were associated with OS in patients with UBTC following palliative treatment with REMS. The CSM score combining sarcopenia and myosteatosis could predict mortality more precisely than either one. Furthermore, they can be used preoperatively to assist in risk stratification, and timely nutritional support, exercise, and medication are needed when necessary.

## Data Availability Statement

The raw data supporting the conclusions of this article will be made available by the authors, without undue reservation.

## Ethics Statement

This study was approved by the Ethics Committee of Zhongda hospital affiliated with Southeast University. All patients in this study were anonymous. The requirement for informed consent has been waived due to the retrospective nature of the current study.

## Author Contributions

J-HG was involved in the study supervision, and study design. QC and XL analyzed and interpreted the data and drafted the manuscript. QC and JL were involved in the critical revision of the manuscript. J-HG and JL participated in the percutaneous stent placement. X-JY, X-PZ, and S-YW were involved in the data collection and regular follow-up. All authors read and approved the final manuscript.

## Funding

This study was supported by the Natural science foundation of Jiangsu Province (BE2020785 and BK20190350), the National Natural Science Foundation of China (81971716, 82001935), and the National Key Research and Development Program (2018YFA0704101). The funders of the study had no role in study design, collection, analysis, and interpretation of date.

## Conflict of Interest

The authors declare that the research was conducted in the absence of any commercial or financial relationships that could be construed as a potential conflict of interest.

## Publisher's Note

All claims expressed in this article are solely those of the authors and do not necessarily represent those of their affiliated organizations, or those of the publisher, the editors and the reviewers. Any product that may be evaluated in this article, or claim that may be made by its manufacturer, is not guaranteed or endorsed by the publisher.
